# Supplementary material for: Interferon gamma release assay tests are associated with persistence and completion of latent tuberculosis infection treatment in the United States: Evidence from commercial insurance data
Source: PLoS One. 2020 Dec 3;15(12):e0243102. doi: 10.1371/journal.pone.0243102 (PMC7714216; doi:10.1371/journal.pone.0243102)
Supplement: S1 Table — (PDF) [file pone.0243102.s001.pdf]

S1 Table: Prescription fill patterns in persons receiving >=6 months of isoniazid in a 12 month period who were not categorized has having completed the 6 month regimen.

Categorization in Table 1	Count	Doses in 12 months	Doses in 9 Months	Percent of Total	Months of INH in Year
6 months of isoniazid received but 6-month treatment regimen not complete	2	180	90	0.3%	6
	1	180	120	0.2%	6
	6	180	150	0.9%	6
7 months of isoniazid received but 6-month treatment regimen not complete	2	210	150	0.3%	7
