# Supplementary material for: Interferon gamma release assay tests are associated with persistence and completion of latent tuberculosis infection treatment in the United States: Evidence from commercial insurance data
Source: PLoS One. 2020 Dec 3;15(12):e0243102. doi: 10.1371/journal.pone.0243102 (PMC7714216; doi:10.1371/journal.pone.0243102)
Supplement: S1 File — (PDF) [file pone.0243102.s002.pdf]

S2 File: Supplemental File #2

Sensitivity Test: An Examination of the Impact of Varying the Categorizations of the Pre-Initiation Testing Pattern Variable

The primary explanatory variable in our study was a categorical variable detailing the pattern of LTBI testing received within the 9 months prior to isoniazid treatment initiation. In the analyses included in the paper this variable was categorized as follows: 1) One TST with no subsequent TSTs or IGRAs, 2) One IGRA with no subsequent TSTs or IGRAs, 3) An initial TST followed by one IGRA, but no additional TSTs or IGRAs, 4) Some other pattern of multiple tests.

The analyses below examine whether our results change if the categorizations are changed. Specifically, this sensitivity test used the following categorizations: 1) One TST with no subsequent TSTs or IGRAs, 2) One IGRA with no subsequent TSTs or IGRAs, 3) An initial TST followed by at least one IGRA, and there may have been additional TSTs or IGRAs subsequent to the initial TST, 4) Some other pattern of multiple tests. In essence, the first two categories were unchanged from the original analyses, but six individuals formerly in category 4 were moved to category 3 -- these individuals had a TST followed by an IGRA, but they also had other additional TSTs and/or IGRAs after the initial TST.

The results of the analyses below are quite similar to those included in the main paper. Regardless of the categorization, IGRAs are associated with both treatment completion and duration. Relative to persons with a TST, persons with one IGRA and persons with an IGRA subsequent to a TST have significantly higher levels of treatment completion and greater treatment persistence.

Conclusion: Our findings are robust to variations in pre-treatment testing pattern categorizations.

S2 Table A: Summary measures of isoniazid treatment completion and persistence by pre-treatment LTBI testing pattern.

Categorization 1	Count	% of Total	# Completing ≥6 Months	# Completing ≥9 Months	Persistence (Median # Months)
One TST	339	51.2%	143 (42.2%)	68 (20.1%)	5
One IGRA	191	28.9%	105 (55.0%)	57 (29.8%)	6
TST followed by one or more IGRAs; additional TSTs may also have been administered	64	9.7%	40 (62.5%)	25 (39.1%)	8
Other pattern of multiple TSTs and/or IGRAs	68	10.3%	39 (57.4%)	23 (33.8%)	6

S2 Table B: Adjusted associations between sample characteristics and level of latent tuberculosis infection treatment completion with isoniazid. This table is comparable to Table 4, the only difference is in how the pre-initiation testing patterns were categorized.

	**Adjusted Odds Ratio (aOR)**	**95% Confidence Interval of aOR**		**p-value**
		**Lower**	**Upper**	
Pre-Treatment Testing Pattern				
One TST	1.00 (base)			
One IGRA	1.59	1.09	2.34	0.017
TST followed by one or more IGRAs; additional TSTs may also have been administered	2.16	1.28	3.65	0.004
Other pattern of multiple TSTs and/or IGRAs	1.72	1.02	2.89	0.040
Sex				
Female	1.00 (base)			
Male	1.09	0.80	1.47	0.591
Age Group				
0-14	1.00 (base)			
15-29	0.62	0.37	1.02	0.061
30-44	0.50	0.30	0.84	0.008
45-64	0.62	0.36	1.06	0.082
Census Region				
Northeast	1.00 (base)			
Midwest	0.87	0.48	1.57	0.635
South	0.87	0.50	1.50	0.610
West	1.14	0.71	1.82	0.583
Patient Location				
Large Central Metro County	1.00 (base)			
Large Fringe Metro County	0.78	0.49	1.23	0.281
Any Smaller County	0.87	0.49	1.54	0.628
% of Households Under FPL in County				
<15%	1.00 (base)			
≥15%	0.54	0.37	0.79	0.001
Insurance Type				
HMO	1.00 (base)			
POS	1.57	0.95	2.61	0.079
PPO	2.93	1.62	5.31	0.000
Supply of Isoniazid Received on Date of 1st Fill	*Neither regimen completed vs. 6 months completed*			
< 2 month supply	1.00 (base)			
>= 2 month supply	1.55	0.84	2.86	0.164
	<9 months completed vs. >=9 months completed			
< 2 month supply	1.00 (base)			
>= 2 month supply	2.99	1.62	5.52	<0.001
Period Regimen Started				
2011 Q3-4	1.00 (base)			
2012 Q1-4	1.14	0.75	1.74	0.525
2013 Q1-4	1.22	0.80	1.87	0.354

2014 Q1	1.99	0.95	4.18	0.068
State TB Rate	0.85	0.72	1.00	0.043
% Foreign Born in County	1.01	0.99	1.03	0.213
Count of Clinical Indicators				
None	1.00 (base)			
1	1.46	1.02	2.08	0.036
2 or more	1.54	0.87	2.75	0.138

S2 Table C: Adjusted associations between sample characteristics and the number of months of isoniazid received in the 1 year after initiation of latent tuberculosis infection treatment. This table is comparable to Table 5, the only difference is in how the pre-initiation testing patterns were categorized.

	Adjusted Incidence Rate Ratio (aIRR)	95% Confidence Interval of aIRR		p-value
		Lower	Upper	
Pre-Treatment Testing Pattern				
One TST	1.00 (base)			
One IGRA	1.14	1.02	1.29	0.027
TST followed by one or more IGRAs; additional TSTs may also have been administered	1.19	1.01	1.40	0.033
Other pattern of multiple TSTs and/or IGRAs	1.14	0.97	1.34	0.107
Sex				
Female	1.00 (base)			
Male	1.05	0.96	1.16	0.284
Age Group				
0-14	1.00 (base)			
15-29	0.88	0.75	1.03	0.099
30-44	0.81	0.69	0.95	0.009
45-64	0.87	0.74	1.03	0.103
Census Region				
Northeast	1.00 (base)			
Midwest	1.01	0.84	1.22	0.921
South	0.96	0.81	1.13	0.593
West	1.05	0.91	1.21	0.532
Patient Location				
Large Central Metro County	1.00 (base)			
Large Fringe Metro County	0.92	0.80	1.06	0.226
Any Smaller County	0.99	0.83	1.18	0.884
% of Households Under FPL in County				
<15%	1.00 (base)			
≥15%	0.84	0.75	0.95	0.004
Insurance Type				
HMO	1.00 (base)			
POS	1.10	0.94	1.28	0.241
PPO	1.30	1.09	1.55	0.004
Supply of Isoniazid Received on Date of 1st Fill				
< 2 month supply	1.00 (base)			
>= 2 month supply	1.21	1.02	1.43	0.028
Period Regimen Started				
2011 Q3-4	1.00 (base)			
2012 Q1-4	0.99	0.87	1.13	0.887
2013 Q1-4	1.00	0.88	1.15	0.944
2014 Q1	1.05	0.83	1.32	0.701
State TB Rate	0.97	0.92	1.02	0.190
% Foreign Born in County	1.00	1.00	1.01	0.165
Count of Clinical Indicators				

None	1.00 (base)			
1	1.15	1.03	1.28	0.013
2 or more	1.09	0.92	1.31	0.322
